# SIRT1 Mediates Effects of FGF21 to Ameliorate Cisplatin-Induced Acute Kidney Injury

**DOI:** 10.3389/fphar.2020.00241

**Published:** 2020-03-10

**Authors:** Qiongzhen Chen, Junfeng Ma, Xiaoning Yang, Qinyao Li, Zhuofeng Lin, Fanghua Gong

**Affiliations:** ^1^College of Life and Environmental Science, Wenzhou University, Wenzhou, China; ^2^School of Pharmacy, Wenzhou Medical University, Wenzhou, China; ^3^Engineering Laboratory of Zhejiang Province for Pharmaceutical Development of Growth Factors, Biomedical Collaborative Innovation Center of Wenzhou, Wenzhou, China

**Keywords:** acute kidney injury, renal tubular injury, fibroblast growth factor 21, sirtuin1, cisplatin

## Abstract

Acute kidney injury (AKI) is a common complication in cancer patients. Kidney function is closely related to patients’ quality of life and tumor prognosis. Cisplatin is a highly effective anti-tumor drug. However, the use of cisplatin is limited by its nephrotoxicity. It has been reported that FGF21 has a renal-protective function, but the mechanisms by which it does so remain unclear. In this study, we show that the expression of FGF21 is significantly upregulated in both *in vitro* and *in vivo* cisplatin-induced AKI models. Administration of recombinant FGF21 to cisplatin-induced AKI mice resulted in significantly decreased blood urea nitrogen (BUN) and serum creatinine levels, as well as significantly reduced protein levels of kidney injury molecule-1 (TIM-1), C-caspase 3, and Bax. H&E-stained kidney sections from cisplatin-induced AKI mice treated with recombinant FGF21 showed a relatively normal renal tissue structure, a reduced number of necrotic sites and vacuolar changes, and decreased casts, suggesting alleviated renal tubular injury. Experiments with an AKI cell model (cisplatin-treated HK-2 cells) yielded similar results as the mouse model; recombinant FGF21 significantly downregulated protein expression levels of TIM-1, C-caspase 3, and Bax. Furthermore, administration of recombinant FGF21 to cisplatin-treated AKI models significantly increased SIRT1 expression, and the beneficial effects of FGF21 on kidney injury were reversed by *SIRT1* knockdown. Collectively, our results suggest that SIRT1 mediates the protective effect of FGF21 on cisplatin-induced kidney injury.

## Introduction

Acute kidney injury (AKI) is a clinical syndrome characterized by an abrupt decline in kidney function, a decrease in glomerular filtration rate, and the accumulation of nitrogenous waste in the kidney (Vanmassenhove et al., 2017). Many factors contribute to the induction of AKI, including drugs, hypovolemia, sepsis, and surgical injury (Motwani et al., 2018). Hence, AKI is widely distributed among clinical departments (Basile et al., 2016). In hospitalized patients, AKI incidence has reached 5–7% and is still rising, with a mortality rate of 40%. AKI is also important risk factor for the development of chronic kidney disease (Coca et al., 2012; Oh et al., 2017).

Cisplatin (CDDP, cis-Diaminodichloroplatinum) is used as a chemotherapeutic drug for the treatment of many different types of cancer. However, about one-third of the tumor patients develop AKI several days after cisplatin treatment. Therefore, the clinical application of cisplatin is frequently limited by its adverse effects (Fernández-Martínez et al., 2016; Kim et al., 2017; Hori et al., 2017; Oh et al., 2017; Li Z. et al., 2018).

Fibroblast growth factor 21 (FGF21) is mainly expressed in the liver, adipose tissue, and pancreatic islet, and is also expressed in muscle and kidney tissues (Nishimura et al., 2000; So and Leung, 2016; Li, 2019). Due to a lack of the typical heparin-binding domain found in the other family members, FGF21 enters the blood circulation through the endocrine system (Fisher and Maratos-Flier, 2016). FGF21 activity depends on its binding to β-Klotho protein and FGF receptors (FGFRs) and the formation of a stable FGF21/β-Klotho/FGFR complex, which regulates the biological effects of downstream signaling molecules (Kuro, 2019). Previous studies showed that serum levels of FGF21 are significantly increased in both AKI and CKD, and FGF21 concentration is correlated with the severity of kidney injury (Li F. et al., 2018; Suassuna et al., 2019). However, numerous studies have demonstrated that FGF21 functions in alleviating inflammation and in the regulation of oxidative stress and autophagy-induced apoptosis (Lu et al., 2019; Suassuna et al., 2019). Recent works also showed that FGF21 significantly reduces urinary albumin levels in type II diabetic mice and alleviates kidney injury (So and Leung, 2016; Zhao et al., 2017).

Sirtuins are NAD-dependent deacetylases that function in multiple biological processes, such as proliferation, DNA repair, mitochondrial energy homeostasis, and anti-oxidation. Mammals contain seven sirtuins (SIRT1–7), located in different subcellular compartments. SIRT1 is one of the most widely studied sirtuins in kidney. It inhibits cell apoptosis, inflammation, and fibrosis by targeting the deacetylation of p53, FoxO1, and NF-κB, and hence functions in protecting cells (Kim and Lee, 2015; Phillipson-Weiner et al., 2016; Wang et al., 2017, 2019; Zhang et al., 2017; Morigi et al., 2018; Sun et al., 2018; Abdolvahabi et al., 2019).

In this study, we found that recombinant human FGF21 (rhFGF21) exerted significant beneficial effects on tubular epithelial cells and alleviated the decline in renal function in cisplatin-induced AKI. In addition, we found that SIRT1 expression was significantly upregulated in renal tubular epithelial cells and renal tissues during the process of cisplatin-induced kidney injury. rhFGF21 treatment further increased the expression of SIRT1, resulting in decreased apoptosis in cells and improved kidney function.

## Materials and Methods

### Cell Culture

Human renal proximal tubule cells (HK-2) were kindly provided by the Stem Cell Bank at Chinese Academy of Sciences. Cells were cultured in DMEM/F-12 media supplemented with 10% fetal bovine serum (FBS, Gibco, United States), 100 U/ml penicilin, and 100 μg/ml streptomycin. HK-2 cells were transfected with lentivirus siRNA against *SIRT1* (LV-SIRT1-RNAi) (Genechem, Shanghai, China) and Control-RNAi (Genechem, Shanghai, China) for 72 h according to the manufacturer’s instructions. HK-2 cells were pretreated with/without rhFGF21 (5 nM; provided by Key Laboratory of Biotechnology Pharmaceutical Engineering, Wenzhou Medical University) for 1 h, and cultured in the presence of cisplatin (20 μM, Sigma-Aldrich, United States). Cells were collected at different time points (12, 18, and 24 h).

### Mice and AKI Model

Healthy C57BL/6 male mice (∼25 g, 8 wk of age, purchased from Shanghai Slac Laboratory Animal Co., Ltd.) were kept at the barrier facility of the Laboratory Animal Centre of Wenzhou Medical University. All animals were kept for 1 week before the experiments started. All animal procedures were carried out in accordance with the principles of the Basel Declaration and Recommendations. Two experiments were conducted. The first experiment consisted of three different groups representing three different time points (24, 48, and 72 h). The experimental mice were randomly divided into three subgroups at each time point, i.e., the control group (NC), cisplatin model group (CIS), and treatment group (FGF21 + CIS) (*n* = 5 in each subgroup). Based on the procedure described by Li F. et al. (2018), we used a high dose (30 mg/kg) of cisplatin in a single intraperitoneal injection to establish the AKI mouse model. The control group was injected with the same volume of normal saline. As for the FGF21 + CIS group, rhFGF21 was dissolved in PBS and stored at 4°C, and mice were pretreated with rhFGF21 at 0.5 mg/kg intraperitoneally 1 h prior to cisplatin injection. rhFGF21 was administered every 24 h based on the *in vivo* elimination rate of rhFGF21 after cisplatin injection, and the same volume of physiological saline was injected into other groups. Mice were euthanized at different time points (24, 48, and 72 h) after cisplatin injection. In the second experiment, we randomly divided the experimental mice into four groups (*n* = 5 per group), i.e., the control group (Ctrl-RNAi), the model group (Ctrl-RNAi + CIS), the treatment group (Ctrl-RNAi + rhFGF21 + CIS), and the SIRT1 knockdown group (LV-SIRT1-RNAi + rhFGF21 + CIS). LV-SIRT1-RNAi or Ctrl-RNAi (1.6 × 10^9^ TU/kg) (Zheng et al., 2017) was administered by tail vein injection 72 h before cisplatin treatment, followed by the same procedure as in the first the experiment. Mice were euthanized 72 h after cisplatin injection. Blood and kidney tissues were collected and used for biochemical and histopathological examinations. Serum creatinine levels were measured using a Creatinine Assay Kit (Sarcosine Oxidase method); blood urea nitrogen (BUN) levels were measured by a Urea Nitrogen Kit (Urease method). The kits were obtained from the Nanjing Institute of Bioengineering (Nanjing, China).

### Immunoblotting

Analysis was performed to measure the protein levels in kidney tissue and HK-2 cells. Protein in total cell lysates was extracted and quantified by BCA Protein Assay. Protein was separated by SDS-PAGE and transferred to a PVDF membrane (Bio-Rad, Hercules). After blocking the membrane in a TBST solution containing 10% (w/v) non-fat milk for 1.5 h at room temperature, different proteins were probed with corresponding antibodies overnight at 4°C. The following antibodies were used: anti-FGF21 (1:1000, Abcam), anti-SIRT1 (1:1000, Abcam), anti-TIM-1 (1:20,000, Abcam), anti-Bax (1:10,000, Abcam), anti-cleaved caspase-3 (1:1000, Abcam), anti-β-Klotho (1:1000, Abcam), anti-β-actin (1:1000, Cell Signaling Technology), and anti-GAPDH (1:1000, Cell Signaling Technology). Subsequently, membranes were washed three times with TBST and then incubated with horseradish peroxidase (HRP)-conjugated secondary antibody for 1 h at room temperature. Protein signals were detected using a Tanon 5200 Chemiluminescence Imaging system (Tanon-5200) and bands were quantified using ImageJ software.

### Histology and Immunohistochemistry

Prior to histological analysis, kidney tissues were fixed in 4% paraformaldehyde for over 24 h, embedded in paraffin, cut into 5 μm sections, stained with hematoxylin and eosin (H&E), sealed with neutral resin, and observed under an optical microscope. Tissue damage and necrosis were assessed by microscopy (×400) in 10–20 randomly selected fields of view. Tissue damage was scored by the percentages of renal tubular injury, cell debris, brush border loss, and cast formation (0, no damage; 1, less than 25% damage; 2, 25–50% damage; 3, 50–75% damage; 4, more than 75% damage).

For immunohistochemistry analysis, 5 μm kidney tissue sections were incubated with anti-FGF21 (Abcam; Cambridge, United Kingdom) and anti-SIRT1 (Abcam; Cambridge, United Kingdom), followed by the secondary antibody (ZSGB-Bio, Beijing, China) and DAB. For each mouse, 10–20 randomly selected fields of view were scanned under an upright microscope. The percentages of positive staining regions of FGF21 or SIRT1 were quantified using Image-Pro 6.0.

### Immunofluorescent Staining

For immunofluorescent staining analysis, cells were fixed with 4% paraformaldehyde and incubated with Triton X-100 (1%) for 15 min. The cells were incubated with anti-FGF21 and anti-SIRT1 overnight at 4°C, incubated with fluorescent-probe-conjugated goat anti-rabbit at room temperature for 1 h, stained with DAPI (Abcam, Cambridge, United Kingdom), and mounted. Images were captured using a laser confocal microscope (Nikon, Ti-E and A1 plus) and quantitatively analyzed using Image-Pro 6.0.

### Apoptosis Assay

Five-micrometer-thick tissue sections were used for TUNEL staining with the apoptosis detection kit (Boster Biological Technology Co., Ltd., Wuhan, China). Briefly, the slides were deparaffinized, rehydrated, and treated with proteinase K (20 mg/ml) for 15 min at room temperature. The slide was preliminarily incubated with labeling buffer (digoxigenin-dUTP) at room temperature for 2 h. Then blocking solution incubated 30 min at room temperature, and added the reaction mixture containing anti-digoxigenin antibody and Fluorescein-Streptavidin (FITC, green), incubated at 37°C for 30 min. DAPI was used for nuclear counterstaining.

The HK-2 cells (1 × 10^6^ cells/well) were seeded on six-well chamber slides. After different treatments, the slides were detected with the apoptosis detection kit (CY3, red). DAPI was used for nuclear counterstaining. TUNEL-positive cells were imaged under a laser confocal microscope (Nikon, Ti-E and A1 plus).

### Statistical Analysis

Data analysis was performed using GraphPad Prism 6.0 software. All data are expressed as mean ± SEM of three independent experiments; the Student’s *t*-test or one-way ANOVA was used for statistical comparisons of different groups. The Pearson test was performed to determine the linear correlation of two variables. *P* < 0.05 indicated a significant statistical difference.

## Results

### Cisplatin Induces HK-2 Cell Apoptosis *in vitro* and AKI *in vivo*

**FIGURE 1:**
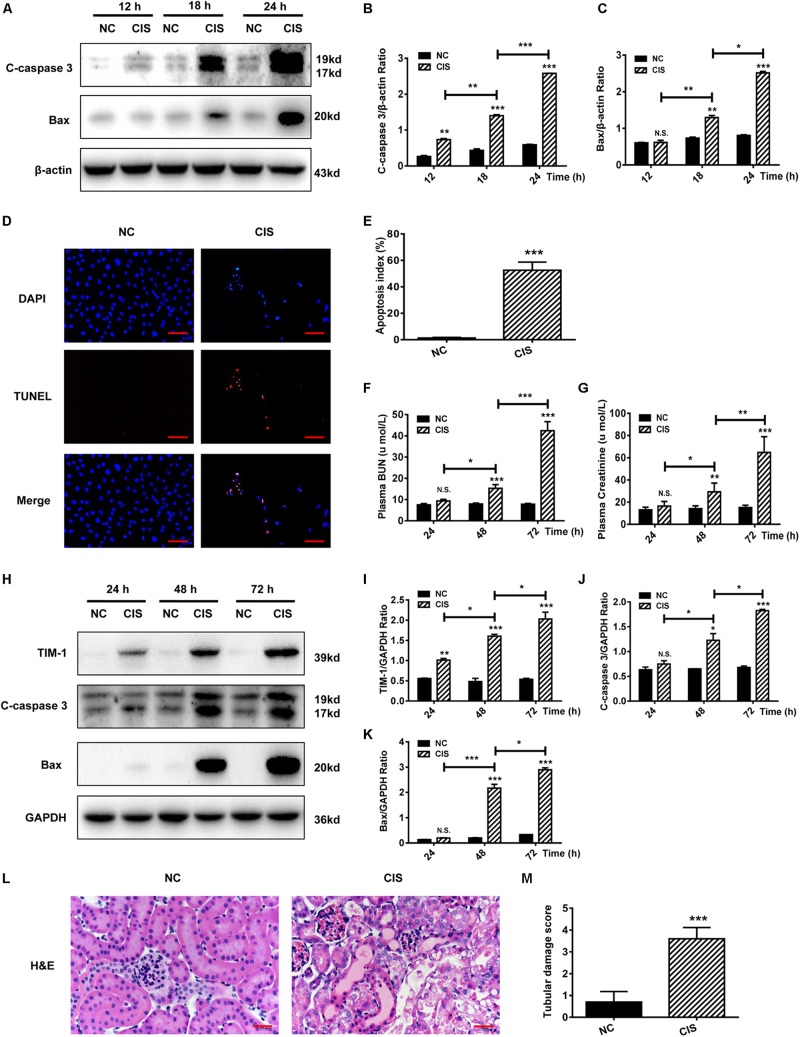
Cisplatin induces tubular cell damage in in vitro and in vivo AKI models. **(A–C)** Representative immunoblotting analysis of C-caspase 3 and Bax in HK-2 cells. Protein levels of C-caspase 3 and Bax were quantified by densitometry, with β-actin as protein loading control. **(D,E)** TUNEL staining in HK-2 cells (scale bar: 100 μm; DAPI: blue, TUNEL: red), and quantitative analysis of TUNEL staining. **(F)** Changes in mouse BUN levels. **(G)** Changes in mouse serum creatinine levels. **(H–K)** Representative immunoblotting analysis of TIM-1, C-caspase 3, and Bax in mouse renal tissue. Expression levels of TIM-1, C-caspase 3, and Bax were quantified by densitometry, with GAPDH as protein loading control. **(L)** H&E staining of mouse renal tissue (scale bar: 100 μm). **(M)** Histopathological scores of H&E staining of mouse kidney tissue. ***P < 0.001, **P < 0.01, *P < 0.05.

HK-2 cells were treated with cisplatin for 12, 18, and 24 h. The expression of the apoptosis-related proteins C-caspase 3 and Bax increased in a time-dependent manner (Figures 1A–C). TUNEL analysis showed nucleus aggregation and significantly increased numbers of apoptotic cells in HK-2 cells after 24 h as compared with control cells (*P* < 0.001, Figures 1D,E and Supplementary Figure S1).

As for *in vivo* experiments in mice, we found that both BUN levels and serum creatinine levels began to rise 24 h after cisplatin treatment, reaching a maximum at 72 h. Both serum creatinine and BUN concentrations at 72 h were about 4.5-fold greater than in the control group, indicating that kidney function was significantly impaired (*P* < 0.001, Figures 1F,G). Analysis of kidney tissue showed that the protein levels of the AKI markers TIM-1, C-caspase 3, and Bax increased significantly in a time-dependent manner (Figures 1H–K). Renal pathological changes were evaluated 72 h after cisplatin administration using H&E staining of mouse kidney paraffin sections. The control group displayed an intact tubular structure, the renal tubules were shaped regularly and arranged neatly, glomerular space was normal without obvious damage, the tubular epithelial cells were arranged in high density, nuclei were round and large, nucleoli were clear and distributed evenly, and no obvious apoptosis and necrosis were found. On the contrary, cisplatin administration resulted in renal tubular damage, necrosis, a large amount of cell debris, vacuolar changes, multiple sites of casts, increased glomerular space, and tissue damage; the average renal tubular injury score was ∼3.5 (Figures 1L,M).

Collectively, these findings indicate that cisplatin causes HK-2 cell apoptosis *in vitro*, and induces AKI *in vivo*, with an impaired tubular structure and a reduced glomerular filtration rate.

### Cisplatin Induces a Significant Upregulation of FGF21 in Renal Tubular Cells *in vitro* and *in vivo*

**FIGURE 2:**
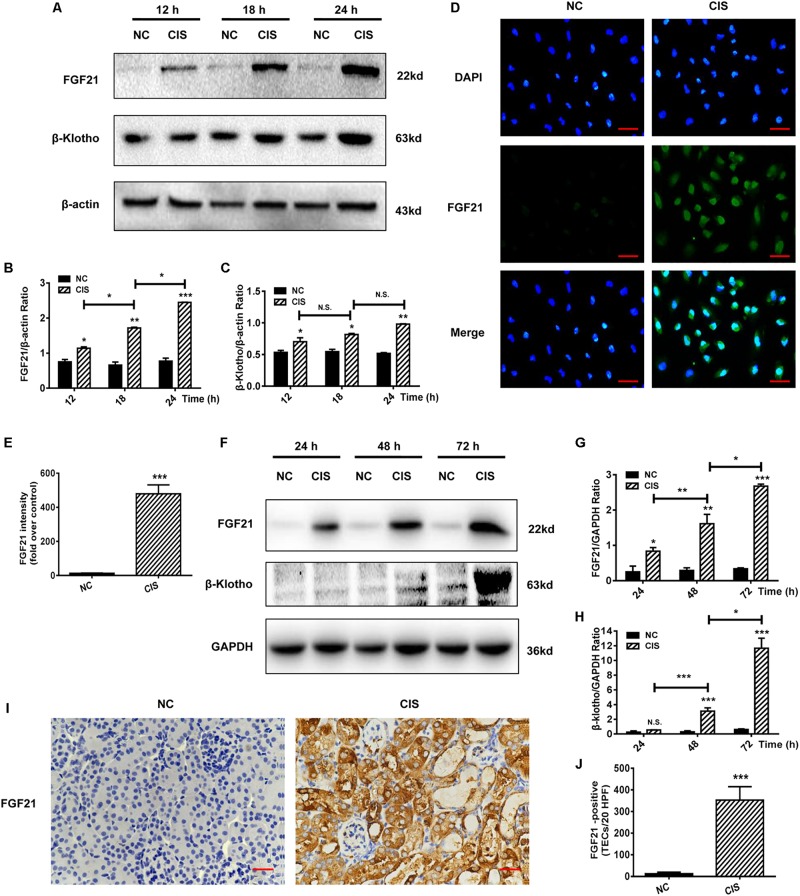
FGF21 is upregulated in *in vivo* and *in vitro* models of renal injury. **(A–C)** Representative immunoblotting analysis of FGF21 and β-Klotho in HK-2 cells. Protein levels of FGF21 and β-Klotho were quantified by densitometry, with β-actin as protein loading control. **(D,E)** Immunofluorescence intensity of FGF21 in HK-2 cells (scale, 100 μm; DAPI: blue, FGF21: green). **(F–H)** Representative immunoblotting analysis of FGF21 and β-Klotho in mouse renal tissue. Expression levels of FGF21 and β-Klotho were quantified by densitometry, with GAPDH as protein loading control. **(I)** Immunohistochemistry staining of FGF21 in mouse renal tissue (scale bar: 100 μm). **(J)** Quantitative immunohistochemistry analysis of FGF21. ***P < 0.001, **P < 0.01, *P < 0.05.

Because FGF21 plays an important role in energy metabolism and cellular stress response, we examined the expression of FGF21 in HK-2 cells after cisplatin treatment. FGF21 expression was significantly upregulated after 12 h; the highest FGF21 levels were observed at 24 h (*P* < 0.001, Figures 2A,B). Expression levels of the FGF21 receptor β-Klotho also increased (Figures 2A,C). Immunofluorescence staining revealed a significant increase in intracellular protein levels of FGF21 after 24 h of cisplatin treatment (*P* < 0.001, Figures 2D,E).

Levels of FGF21 and FGF21 receptor β-Klotho in the AKI mouse model were measured at different time points after cisplatin administration (24, 48, and 72 h). Consistent with the cell culture experiments, *in vivo* FGF21 expression increased in a time-dependent manner, and β-Klotho was significantly upregulated 72 h after cisplatin administration (*P* < 0.001, Figures 2F–H). Immunohistochemistry results showed that 72 h after cisplatin administration, the levels of FGF21 were significantly increased in the area of renal tubular injury, especially in the areas with severe tissue damage or necrosis (*P* < 0.001, Figures 2I,J).

Collectively, our results indicate that cisplatin induces the upregulation of FGF21 expression in renal tubular cells *in vitro* and *in vivo*.

### rhFGF21 Significantly Attenuates Cisplatin-Induced Acute Injury of Renal Tubular Cells *in vitro* and *in vivo*

**FIGURE 3:**
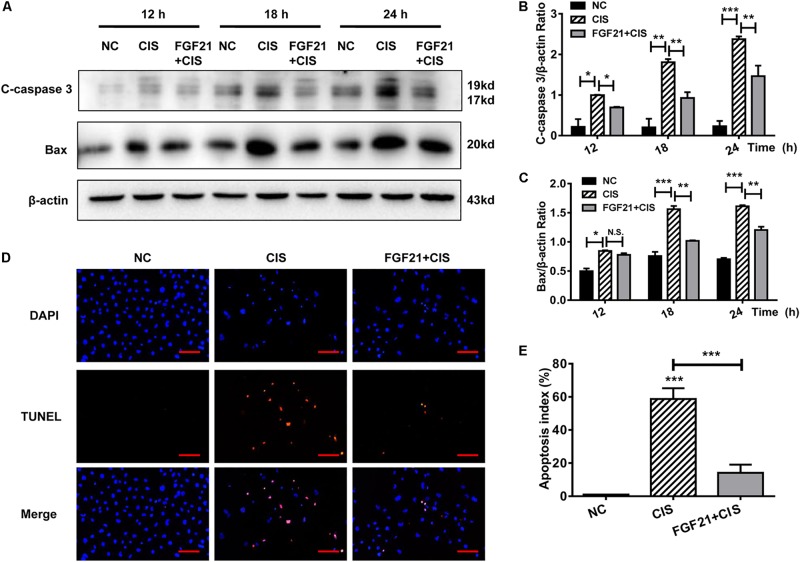
rhFGF21 significantly alleviates HK-2 cell damage. **(A–C)** Representative immunoblotting analysis of C-caspase 3 and Bax in HK-2 cells. Protein levels of C-caspase 3 and Bax were quantified by densitometry, with β-actin as protein loading control. **(D,E)** TUNEL staining in HK-2 cells (scale bar: 100 μm; DAPI: blue, TUNEL: red), and quantitative analysis of TUNEL staining. ***P < 0.001, **P < 0.01, *P < 0.05.

HK-2 cells were pretreated *in vitro* with rhFGF21 for 1 h before cisplatin treatment. We found that rhFGF21 downregulated the expression of C-caspase 3 and Bax differently at different time points (12, 18, and 24 h) compared with cisplatin-treated cells (Figures 3A–C). Moreover, TUNEL staining results indicate that the apoptosis rate of cisplatin-treated HK-2 cells was >50%. In comparison, rhFGF21 pretreatment reduced the percentage of apoptotic cells to less than 20% (Figures 3D,E).

**FIGURE 4:**
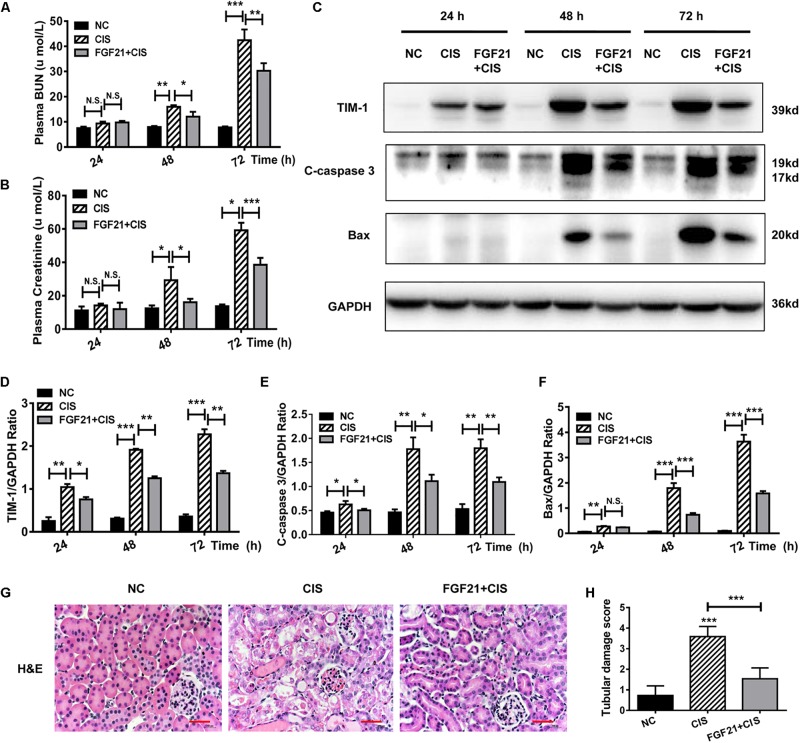
rhFGF21 significantly reduces tubular cell damage in AKI mouse model. **(A)** Changes in mouse BUN levels. **(B)** Changes in mouse serum creatinine levels. **(C–F)** Representative immunoblotting analysis of TIM-1, C-caspase 3, and Bax in mouse renal tissue. Expression levels of TIM-1, C-caspase 3, and Bax were quantified by densitometry, with GAPDH as protein loading control. **(G)** H&E staining of mouse renal tissue (scale bar: 100 μm). **(H)** Histopathological scores of H&E staining of mouse kidney tissue. ***P < 0.001, **P < 0.01, *P < 0.05.

Similarly, we studied the protective effects of rhFGF21 on renal tissues in the mouse AKI model. Our results indicate that at 24 h after rhFGF21 administration, there was no significant difference in BUN and serum creatinine levels among the three groups. At 48 h and later, the administration of rhFGF21 significantly decreased the BUN and serum creatinine levels (Figures 4A,B). In addition, immunoblot analysis demonstrated that mice treated with both cisplatin and rhFGF21 had significantly lower levels of TIM-1, C-caspase 3, and Bax than those treated with cisplatin alone (Figures 4C–F). Renal pathological changes were evaluated 72 h after cisplatin administration by H&E staining of mouse kidney paraffin sections. Microscopic evaluation of the control group showed that the tubular structure was unchanged, tubular epithelial cells were arranged in high density, and no obvious tissue damage was observed. In contrast, intraperitoneal injection of cisplatin resulted in impaired tubular structure, accompanied by increases in glomerular space and casts, a large amount of cell debris, and vacuolar changes, and the average renal tubular injury score was ∼3. rhFGF21 treatment reversed cisplatin-induced renal injury. Although tissue damage was more severe than in the control group, rhFGF21 treatment resulted in a relatively normal tissue structure, fewer necrotic sites and vacuolar changes, and fewer casts as compared with cisplatin-treated mice. The average tissue damage score was reduced to ∼1 by rhFGF21 (Figures 4G,H).

These experimental results demonstrate that rhFGF21 plays a cytoprotective or prosurvival role during cisplatin treatment in HK-2 cells and mouse AKI.

### rhFGF21 Upregulates SIRT1 Expression in Renal Tubular Cells *in vivo* and *in vitro*

**FIGURE 5:**
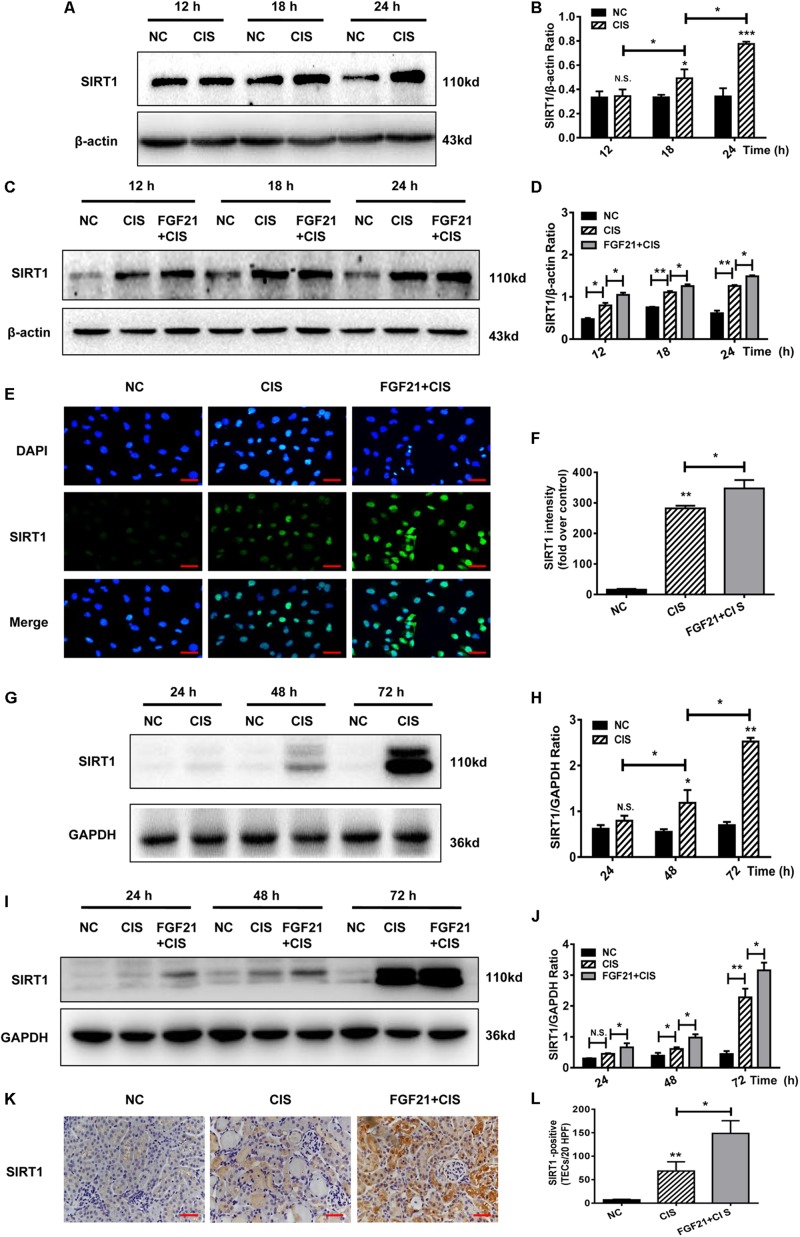
rhFGF21 upregulated SIRT1 expression in renal injury models. **(A–D)** Representative immunoblotting analysis of SIRT1 in HK-2 cells. Protein levels of SIRT1 were quantified by densitometry, with β-actin as protein loading control. **(E,F)** Immunofluorescence intensity of SIRT1 in HK-2 cells (scale, 100 μm; DAPI: blue, SIRT1: green). **(G–J)** Representative immunoblotting analysis of SIRT1 in mouse renal tissue. Expression level of SIRT1 was quantified by densitometry, with GAPDH as protein loading control. **(K)** Immunohistochemistry staining of SIRT1 in mouse renal tissue (scale bar: 100 μm). **(L)** Quantitative immunohistochemistry analysis of SIRT1. ***P < 0.001, **P < 0.01, *P < 0.05.

SIRT1 is a key player in cisplatin-induced AKI and nephrotoxicity (Kim et al., 2019; Zhang et al., 2019). In cisplatin-treated HK-2 cells, SIRT1 protein levels were upregulated, similarly to FGF21 (Figures 5A,B). In cisplatin-treated HK-2 cells, SIRT1 expression was increased, and rhFGF21 further promoted SIRT1 expression (Figures 5C,D and Supplementary Figures S2A–D). Quantification of immunofluorescence signals confirmed that rhFGF21 promotes the expression of SIRT1 (Figures 5E,F).

In the AKI mouse model, SIRT1 expression was not significantly increased at 24 h, but it was increased at 48 h and later (Figures 5G,H). Consistent with our *in vitro* study, we found that rhFGF21 treatment upregulated SIRT1 expression in AKI mice at different time points (*P* < 0.05 at 24 h, Figures 5I,J). Our immunohistochemistry results indicate that cisplatin induced expression of SIRT1 72 h after cisplatin injection, and rhFGF21 further promoted renal SIRT1 expression (Figures 5K,L).

Collectively, these results indicate that rhFGF21 can upregulate SIRT1 expression in injured renal tubular cells in a timely manner. Hence, the underlying mechanisms by which rhFGF21 attenuates cisplatin-induced AKI may involve SIRT1.

### SIRT1 Mediates FGF21-Induced Attenuation of Cisplatin-Induced HK-2 Cell Apoptosis and Mouse AKI

**FIGURE 6:**
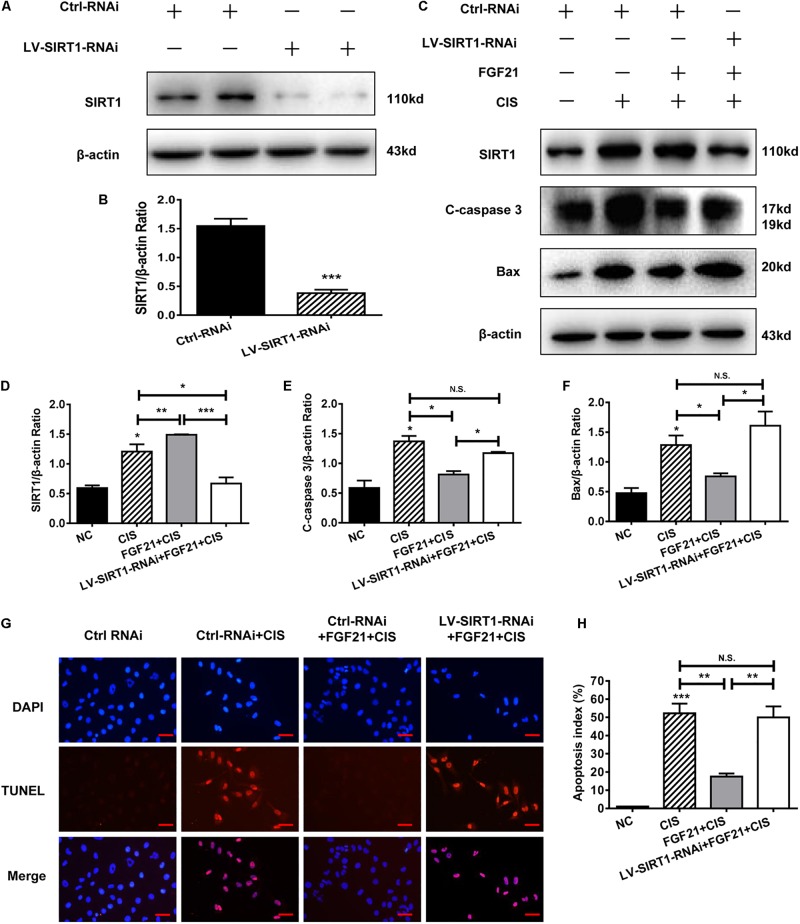
SIRT1 mediates the beneficial effects of FGF21 on cisplatin-induced HK-2 cell damage. **(A,B)** Representative immunoblotting analysis of SIRT1 after LV-SIRT1-RNAi transfection in HK-2 cells. Protein levels of SIRT1 were quantified by densitometry, with β-actin as protein loading control. **(C–F)** Representative immunoblotting analysis of SIRT, C-caspase 3, and Bax after LV-SIRT1-RNAi transfection in HK-2 cells. Protein levels of SIRT1, C-caspase 3, and Bax were quantified by densitometry, with β-actin as protein loading control. **(G,H)** TUNEL staining in HK-2 cells (scale bar: 100 μm; DAPI: blue, TUNEL: red), and quantitative analysis of TUNEL staining. ***P < 0.001, **P < 0.01, *P < 0.05.

SIRT1 plays an important role in the repair of organ and cell damage. Our previous experiments showed that cisplatin induces the expression of SIRT1 in HK-2 cells and mouse kidney tissue. Moreover, rhFGF21 further upregulates the expression of SIRT1 and improves cisplatin-induced AKI. To determine whether rhFGF21 prevents cisplatin-induced AKI by activating SIRT1, we examined the function of rhFGF21 in HK-2 cells after *SIRT1* knockdown. Results indicate that SIRT1 expression was effectively downregulated after LV-SIRT1-RNAi transfection in HK-2 cells. SIRT1 protein levels in HK-2 cells were reduced by approximately 80% after LV-SIRT1-RNAi transfection compared with Ctrl-RNAi (Figures 6A,B).

Next, we knocked down *SIRT1* in HK-2 cells; we found that *SIRT1* knockdown significantly reversed the beneficial effect of rhFGF21 on cisplatin-induced apoptosis (Figures 6C,D). Compared with the rhFGF21 + cisplatin group, *SIRT1* knockdown cells treated with rhFGF21 + cisplatin showed significantly higher expression levels of C-caspase 3 and Bax (Figures 6C,E,F). In cells treated with rhFGF21 + cisplatin, TUNEL analysis showed a significant increase in the percentage of apoptotic cells after *SIRT1* knockdown (Figures 6G,H). These results indicate that the beneficial effects of rhFGF21 on cell damage are significantly decreased in *SIRT1*-knockdown HK-2 cells.

**FIGURE 7:**
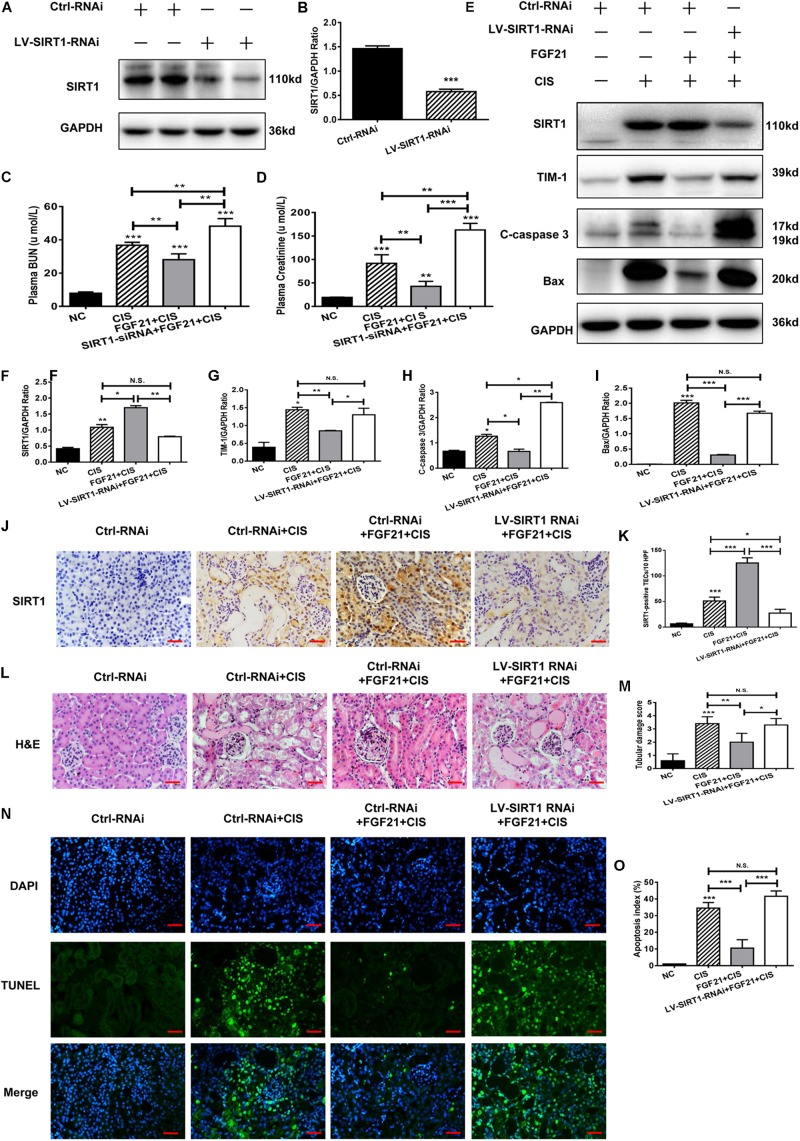
SIRT1 mediates the beneficial effects of FGF21 on cisplatin-induced mouse AKI. **(A,B)** Representative immunoblotting analysis of SIRT1 in *SIRT1* knockdown mice. Protein levels of SIRT1 were quantified by densitometry, with β-actin as protein loading control. **(C)** Changes in mouse BUN levels. **(D)** Changes in mouse serum creatinine levels. **(E–I)** Representative immunoblotting analysis of SIRT1, TIM-1, C-caspase 3, and Bax. Protein levels of SIRT1, TIM-1, C-caspase 3, and Bax were quantified by densitometry, with GAPDH as loading control. **(J)** Immunohistochemistry staining of SIRT1 in mouse renal tissue (scale bar: 100 μm). **(K)** Quantitative immunohistochemistry analysis of SIRT1. **(L)** H&E staining of mouse renal tissue (scale bar: 100 μm). **(M)** Histopathological score of H&E staining of renal tissue. **(N,O)** TUNEL staining of renal tissue (scale bar: 100 μm; DAPI: blue, TUNEL: green), and quantitative analysis of TUNEL staining. ***P < 0.001, **P < 0.01, *P < 0.05.

Similar results were obtained in our *in vivo* experiments. SIRT1 expression in C57BL/6 mice was effectively knocked down after LV-SIRT1-RNAi transfection (Figures 7A,B). In mice treated with rhFGF21 + cisplatin, BUN and serum creatinine levels were significantly increased (Figures 7C,D). When SIRT1 expression was inhibited (Figures 7E,F,J,K), BUN and serum creatinine levels were higher than in rhFGF21 + cisplatin mice (Figures 7C,D). Immunoblot analysis showed that SIRT1 knockdown increased TIM-1, C-caspase 3, and Bax levels in mouse kidney compared with rhFGF21 + cisplatin mice (Figures 7E,G–I). H&E staining showed that rhFGF21 could not effectively ameliorate kidney damage after SIRT1 knockdown, and kidney tissue exhibited tubular structure disorder, a large amount of cell debris, vacuolar changes, increased casts and glomerular space, and aggravated tissue damage (Figures 7L,M). Furthermore, TUNEL analysis was performed using cisplatin-treated mice after SIRT1 knockdown. We found that the percentage of apoptotic cells in renal tissue was higher than 50%, even after rhFGF21 treatment (Figures 7N,O).

In conclusion, these results indicate that *SIRT1* knockdown reduces the protective effects of rhFGF21 on cisplatin-induced AKI both *in vitro* and *in vivo*.

## Discussion

In this study, we discovered that cisplatin induces acute tubular cell injury *in vitro* and *in vivo*. Cisplatin also increases FGF21 and SIRT1 protein levels in kidney. Our results indicate that rhFGF21 administration attenuates cisplatin-induced nephrotoxicity. We further explored the involvement of FGF21/SIRT1 in renal protection.

Two phenomena cause cisplatin-induced AKI, i.e., cisplatin accumulation in tubular cells and renal tubular cell injury (Fernández-Martínez et al., 2016; Oh et al., 2017). Tumor patients are prone to AKI during cisplatin treatment. Their clinical manifestations include a decrease in glomerular filtration rate, an increase in BUN and serum creatinine levels, and electrolyte disorders (Arany and Safirstein, 2003). Our results indicate that both BUN levels and serum creatinine levels began to rise 24 h after cisplatin treatment, reaching a maximum at 72 h in mice. Currently, levels of serum creatinine, BUN, cystatin-C, TIM-1, and NGAL are routinely measured to investigate cisplatin-induced AKI models (Holditch et al., 2019). Our studies also indicate the protein levels of the AKI markers TIM-1, C-caspase 3, and Bax increased significantly in a time-dependent manner.

Previous studies have shown that FGF21 regulates glucose and lipid metabolism to maintain energy balance and metabolic homeostasis. It is mainly expressed in the liver, adipose tissue, and pancreatic islet under physiological conditions (So and Leung, 2016). It has been shown that the serum FGF21 concentration is significantly increased in patients with diabetic nephropathy, chronic kidney disease, and long-term dialysis (Shahbazi et al., 2015; Li Y.Z. et al., 2018). This is possibly related to the decline in kidney function. Studies also showed that FGF21 has beneficial effects on tissue damage caused by metabolic abnormalities and responses to various cellular and metabolic stresses. It was significantly upregulated as an effective catabolic factor in order to antagonize metabolism and energy imbalance (Gómez-Sámano et al., 2017; Luo et al., 2017). In addition, it has been indicated that upregulation of serum FGF21 levels may delay the progression of diabetic nephropathy and chronic kidney disease (Li F. et al., 2018; Suassuna et al., 2019). Many studies have previously shown that loss of FGF21 causes pathologic damage in multiple animal models, including decreased productivity of pancreatic beta cells and insulin sensitivity (Lee et al., 2018; Wanders et al., 2017), promotion of the development of fatty liver in mice followed by aggravation of liver fibrosis (Singhal et al., 2018), and even aggravation of cisplatin-induced AKI (Holditch et al., 2019). Similarly, overexpression of FGF21 or administration of exogenous FGF21 regulates metabolism, controls the stress response, and attenuates organ damage (So and Leung, 2016; Kang et al., 2018; Lu et al., 2019; Suassuna et al., 2019). Our results are consistent with previous reports. Cisplatin induces a significant upregulation of FGF21 in renal tubular cells *in vitro* and *in vivo*. Administration of rhFGF21 *in vitro* and *in vivo* reduced cisplatin-induced tubular cell death, significantly decreased BUN and serum creatinine levels, and downregulated the expression of TIM-1, C-caspase 3, and Bax in kidney tissue. We further have confirmed that increased FGF21 expression during AKI can protect the kidneys.

A previous study demonstrated that FGF21 may protect kidney tubular cells by suppressing P53 (Li F. et al., 2018). Here, we investigated the role of SIRT1 from a new perspective. SIRT1 is a key molecule involved in kidney injury (Kim et al., 2019; Zhang et al., 2019). Our study provides both *in vitro* and *in vivo* evidence to show that SIRT1 expression is upregulated in damaged tubular cells during cisplatin-induced AKI. Previous studies have demonstrated the beneficial effects of SIRT1 in many organs, including the liver (Zhu et al., 2014; Abd Elwahab et al., 2017), heart (Li et al., 2019), bone (Hou et al., 2019), and kidney (Kim et al., 2019). In the ischemia-reperfusion AKI model, SIRT1 targets the deoxyacetylation of p53, FoxO1, and NF-κB to regulate the stress response, inflammation, cell senescence, and apoptosis (Grabowska et al., 2017; Zhang et al., 2017; Abdolvahabi et al., 2019; Wang et al., 2019). In addition, SIRT1-mediated inhibition of tubular cell apoptosis has been shown to be the primary mechanism of renal protection (Liao et al., 2019; Ryu et al., 2019; Zhang et al., 2019). Our data also suggest that such a mechanism is important during cisplatin-induced AKI.

Recently, pharmacologic studies have suggested that FGF21 improves cardiac function and alleviates Ang II-induced cardiac hypertrophy in a SIRT1-dependent manner (Li et al., 2019). Similarly, studies have reported that the anti-senescence effect of FGF21 on human umbilical vascular endothelial cells (HUVECs) is SIRT1-dependent (Yan et al., 2017). These findings imply that SIRT1 may contribute to the beneficial effects of FGF21. However, further experiments are needed to verify this hypothesis in cisplatin-induced AKI. In the present study, we demonstrate that rhFGF21 induces the expression of SIRT1, further attenuates renal tubular cell injury, and inhibits apoptosis. Moreover, the renal protective effects of rhFGF21 are decreased upon SIRT1 knockdown, suggesting that SIRT1 mediates the beneficial effects of FGF21 on cisplatin-induced AKI.

In summary, our results demonstrate that FGF21 ameliorates cisplatin-induced nephrotoxicity through SIRT1, preventing kidney failure and tubular cell damage.

## Data Availability Statement

The datasets generated for this study are available on request to the corresponding author.

## Ethics Statement

All animal experimentation was carried out in accordance with the principles of the Basel Declaration and recommendations of the Laboratory Animal Guidelines for Ethical Review of Animal Welfare, Laboratory Animal Ethics Committee of Wenzhou Medical University. The protocol was approved by the Laboratory Animal Centre of Wenzhou Medical University.

## Author Contributions

QC, JM, XY, and QL performed experiments. FG and ZL coordinated the study and oversaw all experiments. FG wrote the manuscript. All authors discussed the results and commented on the manuscript.

## Conflict of Interest

The authors declare that the research was conducted in the absence of any commercial or financial relationships that could be construed as a potential conflict of interest.

## Supplementary Material

FIGURE S1 TUNEL staining in HK-2 cells at different time points. HK-2 cells were treated with cisplatin for 12 h, 18 h, and 24 h. TUNEL staining in HK-2 cells (scale bar: 100 μm; DAPI: blue, TUNEL: red).

FIGURE S2 rhFGF21 upregulated SIRT1 expression in HK-2 cells. **(A–D)** Representative immunoblotting analysis of SIRT1 in HK-2 cells. Protein levels of SIRT1 were quantified by densitometry, with β-actin as protein loading control. ****P* < 0.001, ***P* < 0.01, **P* < 0.05.

## Abbreviations

AKI: acute kidney injury
CIS: cisplatin
kidney injury molecule-1: TIM-1
SIRT1: sirtuin1
FGF21: fibroblast growth factor 21
CKD: chronic kidney disease
NC: negative control
BUN: blood urea nitrogen
C-caspase 3: cleaved caspase-3 .
