# Supplementary figures and images for: SIRT1 Mediates Effects of FGF21 to Ameliorate Cisplatin-Induced Acute Kidney Injury
Source: Front Pharmacol. 2020 Mar 10;11:241. doi: 10.3389/fphar.2020.00241 (PMC7076185; doi:10.3389/fphar.2020.00241)

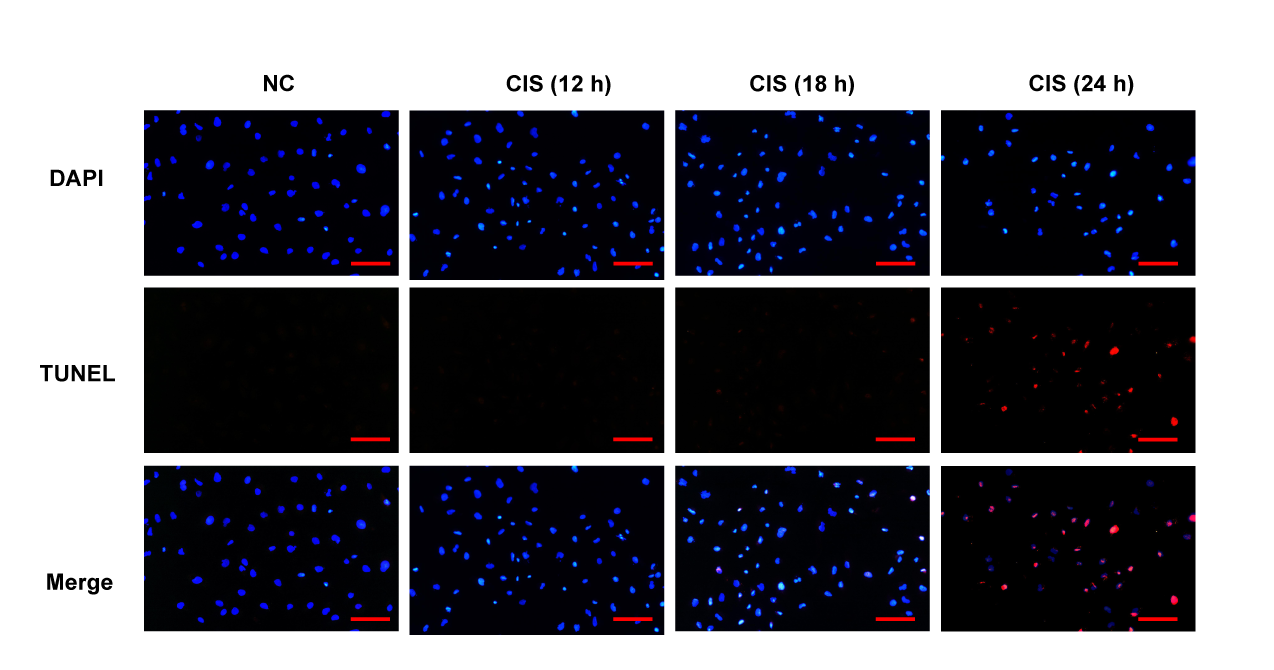

Supplement: FIGURE S1 — TUNEL staining in HK-2 cells at different time points. HK-2 cells were treated with cisplatin for 12 h, 18 h, and 24 h. TUNEL staining in HK-2 cells (scale bar: 100 μm; DAPI: blue, TUNEL: red). [file Image_1.tif]

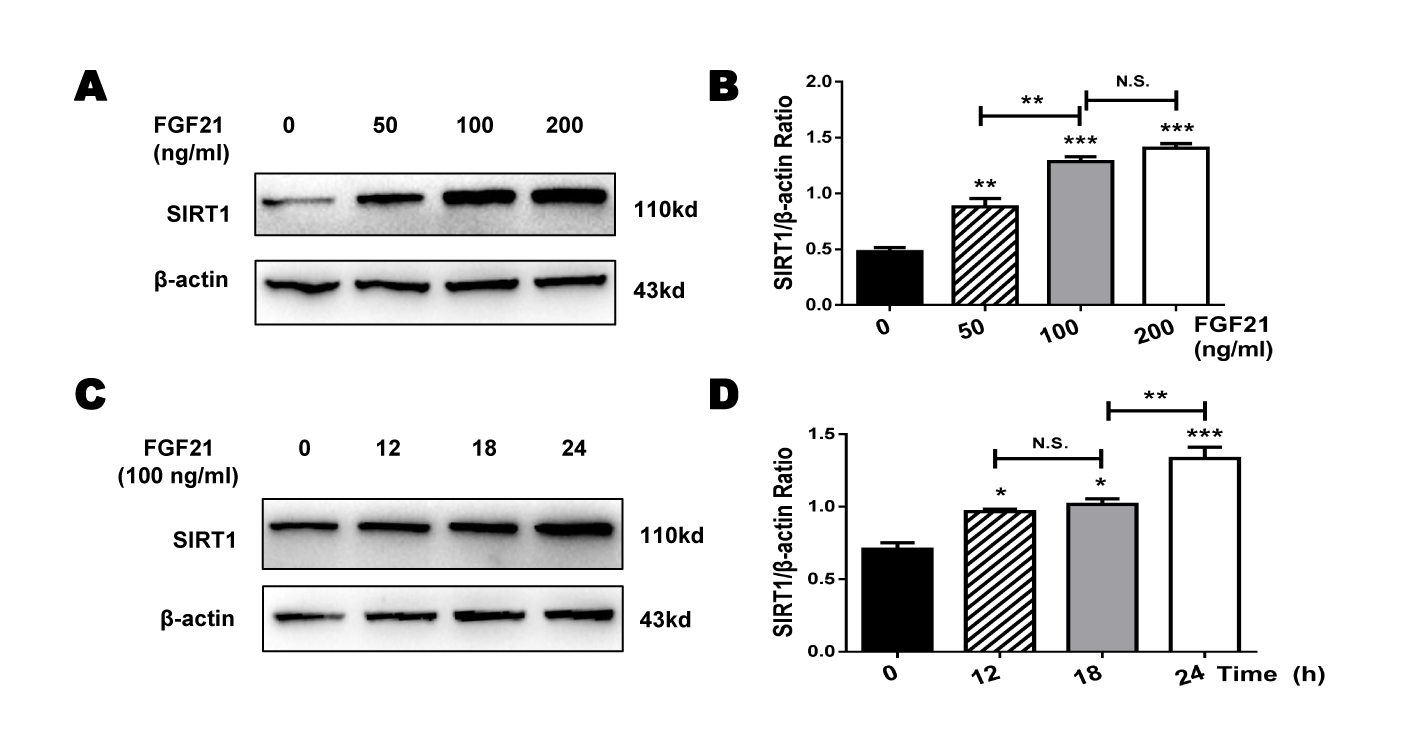

Supplement: FIGURE S2 — rhFGF21 upregulated SIRT1 expression in HK-2 cells. (A–D) Representative immunoblotting analysis of SIRT1 in HK-2 cells. Protein levels of SIRT1 were quantified by densitometry, with β-actin as protein loading control. ***P < 0.001, **P < 0.01, *P < 0.05. [file Image_2.tif]
